# Biodegradation of BTEX by Bacteria Isolated From Soil Contaminated With Petroleum Sludge and Liquid and Solid Petrochemical Effluents

**DOI:** 10.1002/mbo3.70132

**Published:** 2025-11-09

**Authors:** Zahra Mahmoodi Panah, Maryam Jalili Tabaii, Masumeh Sadat Shahidi Rizi

**Affiliations:** ^1^ Department of Microbiology, Faculty of Biological Sciences and Technology Shahid Ashrafi Esfahani University Isfahan Iran

**Keywords:** biodegradation, BTEX, gas chromatography, petrochemical effluents, petroleum sludge

## Abstract

BTEX is a significant pollutant of soil and water. This study aimed to isolate BTEX‐decomposing bacteria from oil sludge‐contaminated soil in the Bahregan area, as well as from liquid and solid petrochemical effluents in Lordegan. The research further investigated the extent of BTEX decomposition by the selected bacteria. The isolated bacteria were screened for their ability to grow and tolerate BTEX, leading to the selection of three strains (G2, M2, and K). The selected bacteria were then exposed to BTEX, and their degradation efficiency was evaluated. For the initial evaluation, residual BTEX was extracted using dichloromethane as the solvent, and the optical density at 600 nm (OD₆₀₀) of the separated organic phase was measured. Subsequently, gas chromatography (GC) analysis was performed for a more accurate assessment. The results from both tests indicated that the highest level of BTEX biodegradation by the tested isolates followed this order: G2(*Arthrobacter pascens*) > M2 (*Bacillus sp*.) > K. The biodegradation rate of each BTEX compound varied by isolate: G2 bacteria: T > B > X > E, M2 bacteria: B > T > X > E, K bacteria: E > X > T > B. Finally, the top‐performing isolates were identified through gene sequencing. The G2 bacteria showed 98.36% similarity to *Arthrobacter pascens B46W*, while the M2 bacteria had 99% similarity to *Bacillus sp. strain jrb‐12w*.

## Graphical abstract

BTEX accounts for a significant proportion of total VOCs emitted from petrochemical facilities. This study was conducted to isolate and identify BTEX‐degrading bacteria from soil contaminated with oil sludge and petrochemical wastewater from Lordegan, followed by evaluating the degradation percentage of this compound by the isolated microorganisms using gas chromatography.

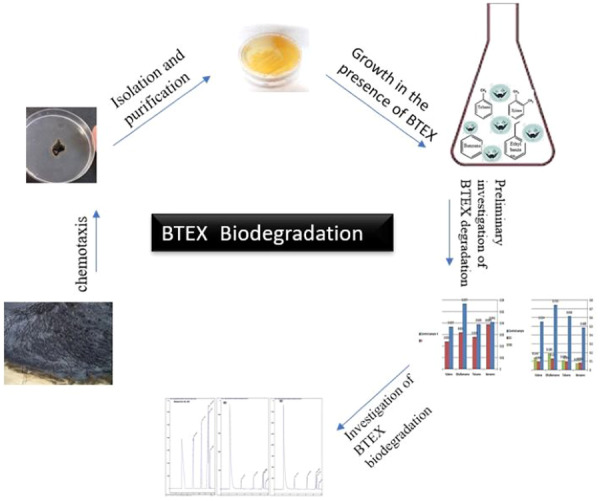

## 1 Introduction

BTXE is a group of volatile organic compounds that contains Benzene, Toluene, Ethylbenzene, and Xylene. These compounds are naturally found in crude oil and its derivatives (gasoline, diesel), jet fuel, and paints. BTEX is commonly utilized in manufacturing plastics, paints, synthetic fibres, and pesticides. BTEX compounds constitute a high percentage of the total volatile organic compounds in petrochemical plants and gasoline pollutants. BTEX accounts for a significant proportion of total VOCs emitted from petrochemical facilities and gasoline‐related pollutants. They are among the most pervasive contaminants of soil and groundwater and are recognized as priority pollutants by the United States Environmental Protection Agency (U.S. EPA) (Singh and Celin 2010; Khodaei et al. 2017; Mohebbi et al. 2024). Environmental release of BTEX can pose serious health risks because it can easily enter the human body through ingestion, inhalation, or dermal exposure. Long‐term exposure to BTEX causes neurological disorders, anaemia, excessive bleeding, and leukaemia (Singh and Celin 2010; Wongbunmak et al. 2020). Due to its high solubility in water, BTEX readily contaminates groundwater. Primary sources of water pollution include petroleum products, including gasoline and diesel fuel, and crude oil tank leaks (Singh and Celin 2010; Fayemiwo et al. 2017). BTEX release into the atmosphere also causes stratospheric ozone depletion, the greenhouse effect, and global warming (Usman et al. 2020; Kaur et al. 2025). Removing BTEX compounds from the ecosystem has led researchers to find new methods and technologies with high efficiency, low cost, and good performance (Mello et al. 2019; Nordin et al. 2025). The methods developed to remove aromatic compounds in contaminated sites include biological methods (Bioventing and bioremediation), chemical methods (chemical oxidation and soil washing), and physical methods (soil steam extraction and thermal treatment) (Chen et al. 2022a). Today, biological methods, such as microbial biodegradation, have emerged as the most, cost‐ effective, and environmentally sustainable solution for cleaning up BTEX contaminants from polluted sites. In situ bioremediation of aromatic hydrocarbons by native microorganisms is quite feasible. Bacteria can utilize BTEX as a carbon source and degrade it (Mello et al. 2019; Nordin et al. 2025; Chen et al. 2022b; Handayani et al. 2019). Previous research indicate that indigenous microorganisms in groundwater and contaminated soil exhibit high degradation activity. Bacteria can directly interact with pollutants to biodegrade hydrocarbon compounds. The preference of a bacterial strain for a particular hydrocarbon ensures the selection of a spectrum of degradation, and since hydrocarbon compounds have similar structures, a strain's ability to degrade one compound often indicates its potential to degrade other related hydrocarbons (Singh and Celin 2010). Aerobic BTEX‐degrading bacteria are widely distributed in nature. The aerobic biodegradation of monoaromatic compounds follows different metabolic pathways based on the enzymatic capabilities of the microorganisms (Usman et al. 2020; Kaur et al. 2025). Since benzene, toluene, ethylbenzene, and xylene are often all found together in contaminated sites, microbial strains capable of simultaneously degrading all BTEX components are more favourable than microorganisms targeting individual compounds (Singh and Celin 2010). Today, extensive research is being conducted to identify bacterial strains that can convert pollutants into harmless byproducts. These bacteria offer an environmentally friendly method for removing environmental pollutants (Handayani et al. 2019). This study was conducted to isolate and identify BTEX‐degrading bacteria from soil contaminated with oil sludge and petrochemical wastewater from Lordegan, followed by evaluating the degradation percentage of this compound by the isolated microorganisms using gas chromatography. Finally, based on the results, the most effective bacteria with the highest biodegradation power were selected to clean the contaminated areas.

## 2 Materials and Methods

### 2.1 Sample Collection

Soil contaminated with oil sludge from the Bahrgan area, as well as liquid and solid petrochemical waste from Lordegan was collected under sterile conditions and promptly transported to the laboratory.

### 2.2 Isolation and Selection of BTEX‐Degrading Bacteria

A synthetic agar semi‐solid mineral medium was used to isolate alkane‐degrading bacteria from oil sludge. The medium contained the following components (g/L): K_2_HPO_4_, 1; KH_2_PO_4_, 1; (NH_4_)_2_SO_4_, 1; MgSO_4_, 0.04; and FeCl_3_, 0.004, along with 10% Persian Gulf seawater and 0.75% agar. The pH was adjusted to 7. The medium was spread onto a plate, and wells were cut into it. Contaminated soil containing oil sludge, along with both solid and liquid petrochemical wastes, was placed in the central well; at the same time separate carbon sources, including benzene, toluene, ethylbenzene, and xylene, were added to the surrounding wells. The samples were incubated at 30°C for 3 weeks. After incubation, chemotaxis was studied, and the bacteria that grew between the central well and the surrounding wells containing carbon sources were isolated and purified. Chemotaxis refers to the movement of bacteria from a sample toward a carbon source, which facilitates their growth along and around the initial carbon source. In addition to helping isolate bacteria in the shortest possible time, chemotaxis can also indicate the suitability of the carbon source (Shahidi Rizi et al. 2017).

### 2.3 Growth and Tolerance in the Presence of Benzene, Toluene, Ethylbenzene, and Xylene

The isolated bacteria were cultured spirally in a solid mineral base medium containing 1% of each carbon source alone (Benzene, Toluene, Ethylbenzene, and Xylene). Cultured plates were incubated at 30°C for 48 h. Finally, based on the turbidity of the culture layer, the results were reported from negative to four positives. Plates with three or more positive results were selected for further analysis (Jawhari 2014; Al‐Zahrani et al. 2022).

### 2.4 Growth Studies in the Presence of 1% BTEX

In this step, a pre‐culture with a turbidity equal to half McFarland was prepared from each of the bacteria selected in the previous step. Then 2% bacterial pre‐culture and 1% BTEX mixture (ratio 1:1:1:1) were added to the liquid mineral base culture medium and incubated for 12 days in a shaker incubator at 30°C with a shaking speed of 140 rpm. Bacterial growth was monitored every 3 days by measuring optical density at 600 nm (OD_600_) and quantifying bacterial colony‐forming units (CFU) on nutrient agar (Singh and Celin 2010; Handayani et al. 2019).

### 2.5 Preliminary Investigation of BTEX Degradation

For this purpose, each isolated and selected bacterium was cultured in a mineral‐based medium containing 1% BTEX for 12 days. The bacterial culture samples were then mixed with dichloromethane in a 1:1 ratio and centrifuged at 50,000 rpm for 5 min. The aqueous and organic phases were subsequently separated, and the absorbance of the organic phase was measured with a spectrophotometer at a wavelength of 600 nm. Finally, the bacteria that exhibited the greatest ability to reduce OD_600_ in the presence of dichloromethane were selected for further studies (Usman et al. 2020).

### 2.6 Investigation of BTEX Biodegradation

The gas chromatography method was employed to accurately determine the extent of BTEX degradation by bacteria selected in the previous step. Each bacterium was cultivated separately in screw‐cap bottles containing a mineral base culture medium with 1% BTEX. The bottles were then placed in a shaker incubator at 30°C and 140 rpm for 12 days. A control bottle containing the culture medium without bacterial inoculation was also included. The decomposition of BTEX compounds was subsequently analyzed through chromatographic techniques. To prepare the sample, 1 ml of dichloromethane extraction solvent was added to 10 ml of the bacterial culture, followed by extraction. Finally, the extracted organic phase was injected into the gas chromatography system, specifically the Agilent Technologies Model 6890 N. The chromatographic analysis was conducted under the following conditions: The temperature program started at an initial temperature of 40°C, and was maintained for a minimum of 5 min. The temperature was then increased at a rate of 10°C per minute until it reached 150°C. Following this, the temperature was ramped further at a rate of 15°C per minute until the final temperature of 240°C was attained, which was held constant for 5 min. The injection port temperature was set at 250°C, and a sample injection volume of 1 microliter was used. The carrier gas was delivered at a flow rate of 2 mL/min, and detection was performed using a flame ionization detector (FID) operating at 280°C. The GC‐MS was equipped with a CP‐Wax 57 CB column of 25 m × 0.32 mm × 1 µm (Miri et al. 2022).

### 2.7 Genetic Identification of BTEX Decomposers

The genomic DNA of selected strains was obtained by boiling. Forward primer 27 F (5‐AGAGTTTGATCCTGGCTCAG‐3) and universal reverse primer 1492 R (5‐TACGYTACCTTGTTACGACTT‐3) were used to amplify the 16SrRNA gene. The PCR program consisted of one cycle at 95°C for 2 min, followed by 30 cycles of 95°C for 30 s, 55°C for 1 min, and 72°C for 1 min, concluding with an extension cycle at 72°C for 10 min. After observing the relevant bands, the isolated bacteria were identified by analyzing the nucleotide sequences using the BLAST program (Shahidi Rizi et al. 2017).

## 3 Results

### 3.1 Isolation and Growth of Bacteria in the Presence of Carbon Sources

Through chemotaxis, we isolated and purified 12 bacterial strains. The findings from the studies on these strains, based on the carbon source toward which they migrated, their morphological characteristics, and their arrangement, are presented in Table 1. We individually evaluated the growth of the isolated bacteria in the presence of each carbon source (benzene, toluene, ethylbenzene, and xylene) by assessing growth density on solid media through visual observation. The results, ranging from the highest turbidity (+4) to the lowest turbidity (+1) and no growth (−), are reported in Table 2. Out of the 12 isolated strains, 7 (M1, M2, M3, G1, G2, G3, K) exhibited strong growth and tolerance to all four carbon sources, achieving growth density scores higher than +3 on solid culture media. Although these bacteria showed chemotaxis toward a specific carbon source, their robust growth in the presence of all four BTEX compounds led to their selection for further studies.

**Table 1:** Morphological characteristics, arrangement and Gram reaction of isolated bacteria and the carbon source to which the bacteria have moved.

Isolated bacteria	Carbon source	Morphology	Gram reaction	Arrangement
M1	B	coccus	+	Irregular clusters
M2	X	Bacilli	+	Chains
M3	X	Bacilli	+	Single
G1	T	Bacilli	+	Single
G2	E	Rod–coccus	+	Diploid—Single
G3	E	Bacilli	+	Single
K	E	Filamentous		Straight filamentous
L1	B	Coccus	+	Irregular clusters
L2	T	Bacilli	+	Chains
L3	E	Coccus	+	Irregular clusters
L4	X	Coccus	+	Irregular clusters
L5	X	Bacilli	+	Irregular clusters

**Table 2:** Investigating the tolerance and growth of isolated bacteria in the presence of each carbon source alone.

Isolated bacteria	B	T	E	X
M1	+4	+3	+3	+3
M2	+4	+4	+4	+4
M3	+4	+4	+4	+4
G1	+4	+4	+3	+3
G2	+3	+4	+4	+4
G3	+4	+4	+4	+4
K	+4	+4	+4	+4
L1	+4	—	—	+1
L2	+2	+3	+2	+4
L3	+2	+1	+3	+1
L4	+1	—	+3	+1
L5	+2	+3	+2	+4

Abbreviations: B, Benzene, E, Ethylbenzene; T, Toluene; X, Xylene.

### 3.2 Bacterial Growth in the Presence of 1% BTEX Mixture

**Figure 1:**
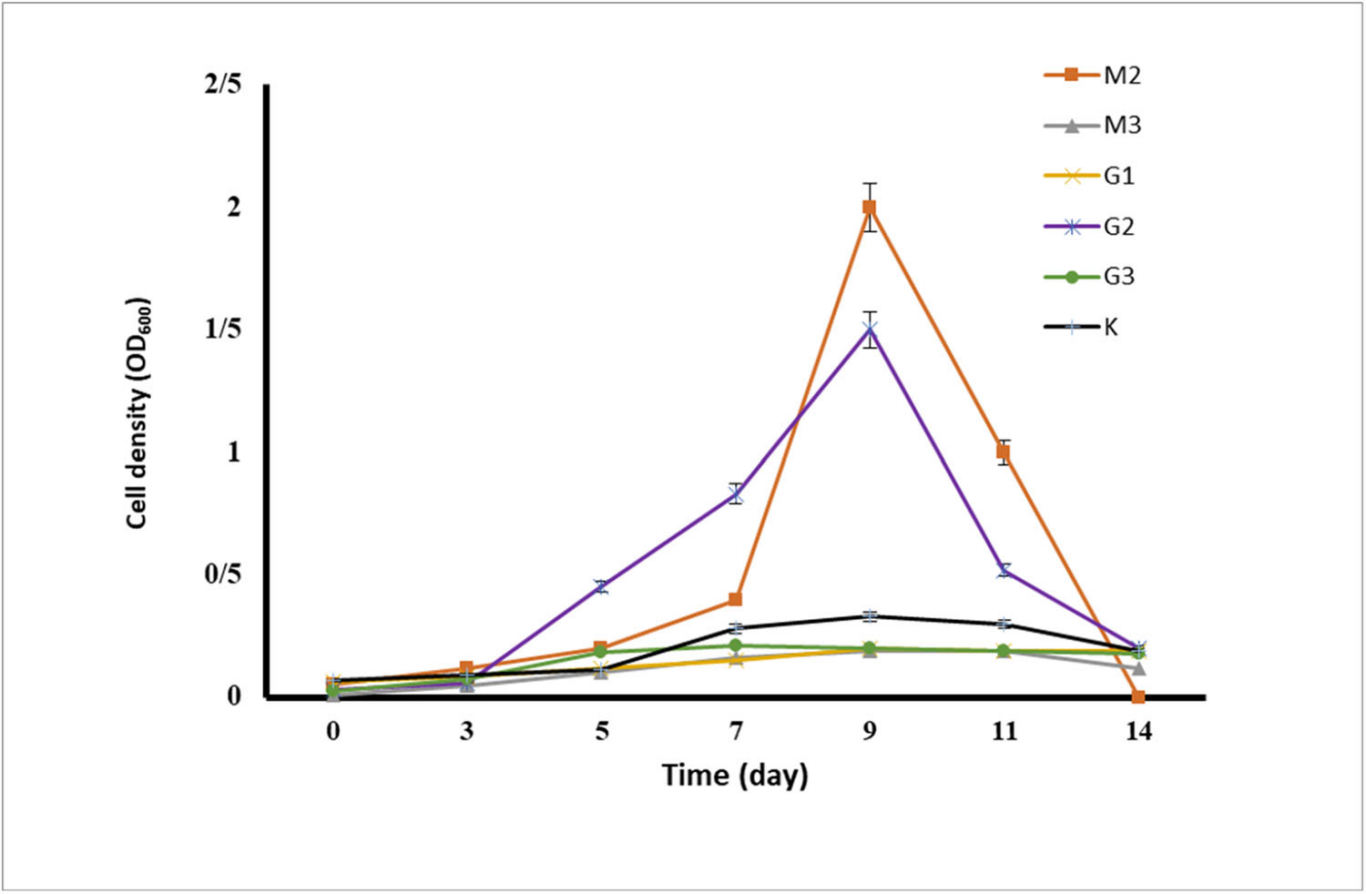
The growth chart of isolated bacteria in the mineral base culture medium in the presence of 1% BTEX using the turbidity method. The results are repeated three times.

**Figure 2:**
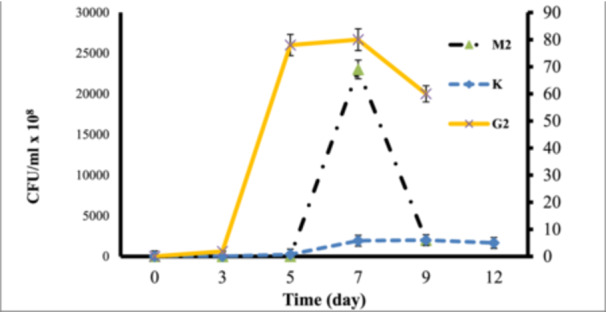
The growth chart of isolated bacteria in the mineral base culture medium in the presence of 1% BTEX using the colony counting method. The results are repeated 3 times.

The results of the growth evaluation for the seven selected bacteria in a liquid medium containing the BTEX mixture are presented in Figure 1, based on turbidity measurements and OD600 readings. As shown in Figure 1, the strains M2, G2, and K exhibited the highest cell densities in the presence of the BTEX mixture. To confirm the growth of these three strains in the 1% BTEX medium, colony counting assays were also performed, with the results displayed in Figure 2. Both turbidity measurements and colony counts indicated that these bacteria had the highest growth rates and cell densities in the presence of BTEX, in the following order: M2 > G2 > K. Consequently, these three strains were selected for further studies to evaluate their potential for BTEX biodegradation.

### 3.3 Determination of BTEX Removal by Isolated Bacteria

**Figure 3:**
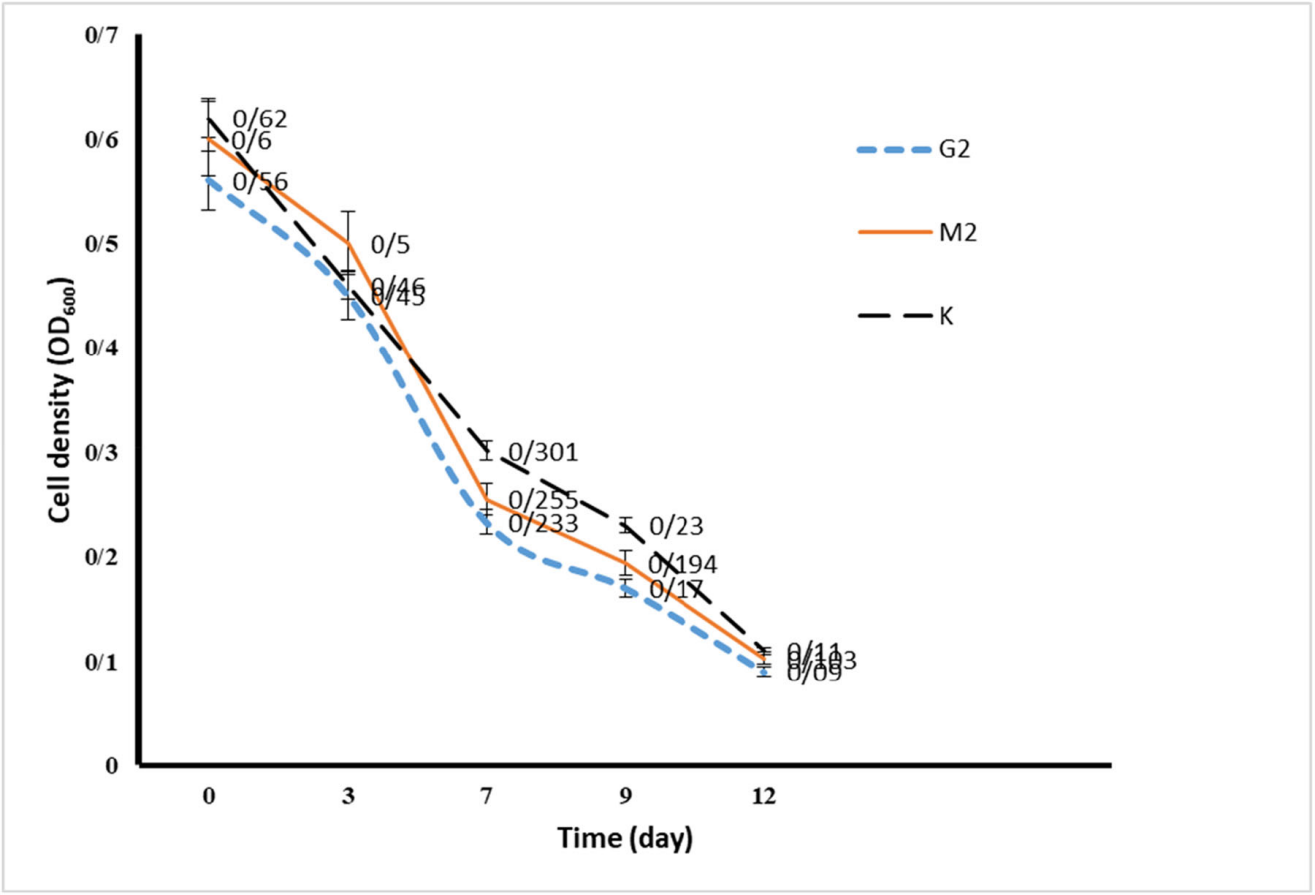
Results of biological reduction of BTEX by bacteria through OD_600_ reading in the organic phase separated from bacterial culture. The results are repeated three times.

The efficiency of BTEX removal by the three bacterial strains selected in the previous stage was evaluated by cultivating them in the presence of BTEX. Residual BTEX was extracted using dichloromethane solvent at 3‐day intervals over 12 days, and the OD_600_ of the separated organic phase was measured. The results are presented in Figure 3. Initially, the OD_600_ was high due to the presence of BTEX in the culture medium. However, as the selected bacteria grew over consecutive days, a decrease in OD_600_ was observed, indicating a reduction in BTEX levels and its biodegradation by the bacteria. Consequently, all three bacterial strains demonstrated the ability to remove BTEX and were selected for further investigation.

### 3.4 Investigating the Biodegradation of BTEX Through Chromatography

**Figure 4:**
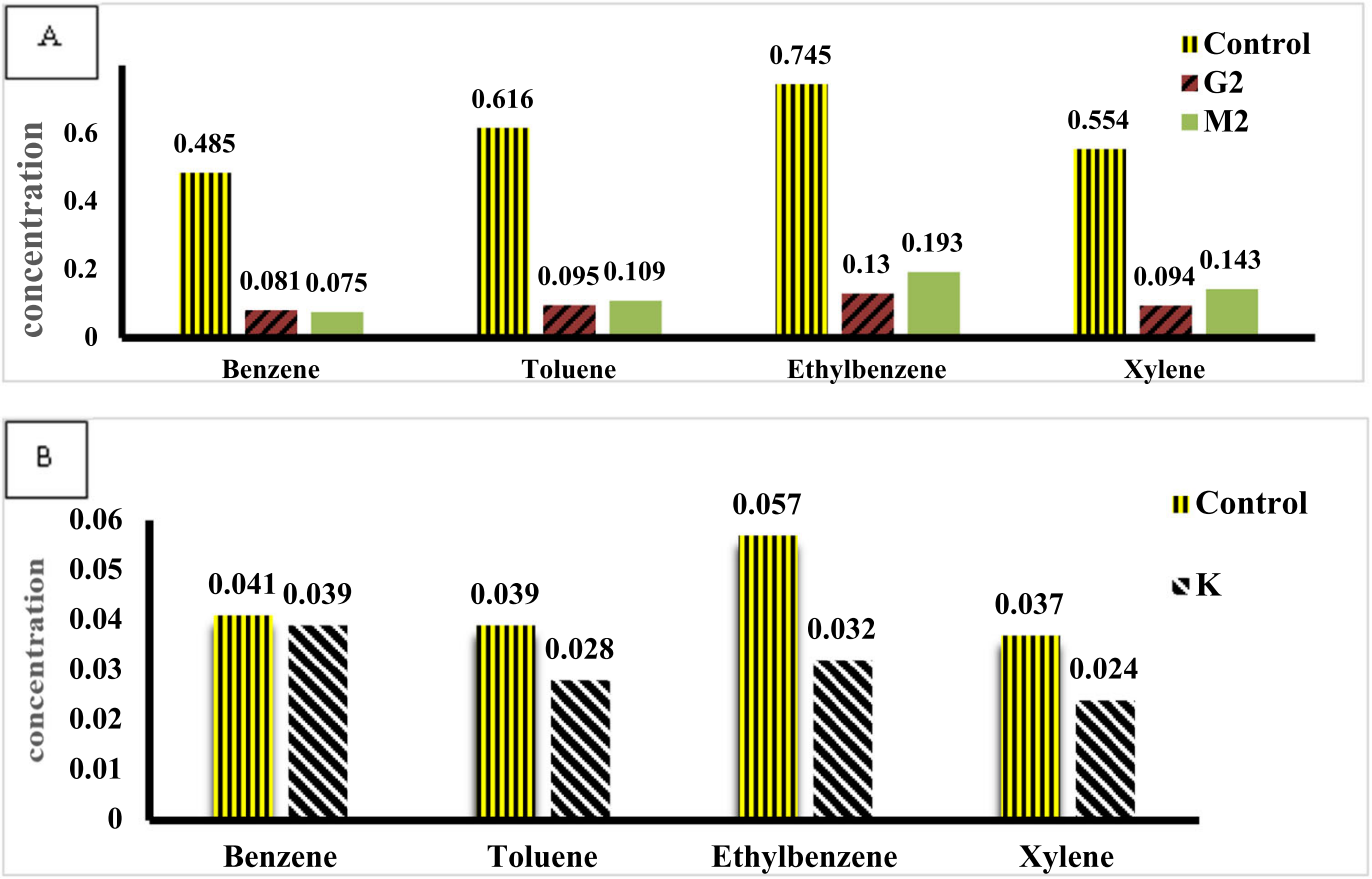
Results of BTEX Biodegradation Based on GC Analysis Using the GC‐FID Method. (A) Residual BTEX concentration in samples treated with bacteria strains G2 and M2 alongside the control sample. (B) Residual BTEX concentration in samples treated with bacterium K alongside the control sample. The best result and the highest reduction of BTEX were achieved by bacterium G2.

**Figure 5:**
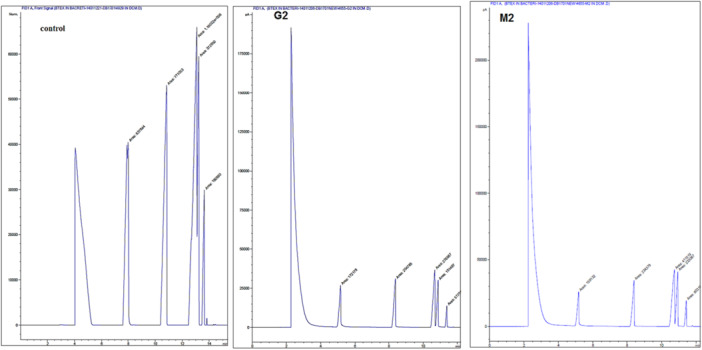
Chromatogram results from the biodegradation of BTEX by G2, M2 bacteria along with the control sample, through GC chromatography analysis based on the GC‐FID test method.The order of the peaks in the mentioned test, according to the column used, includes benzene, toluene, ethylbenzene, and total xylenes.

**Figure 6:**
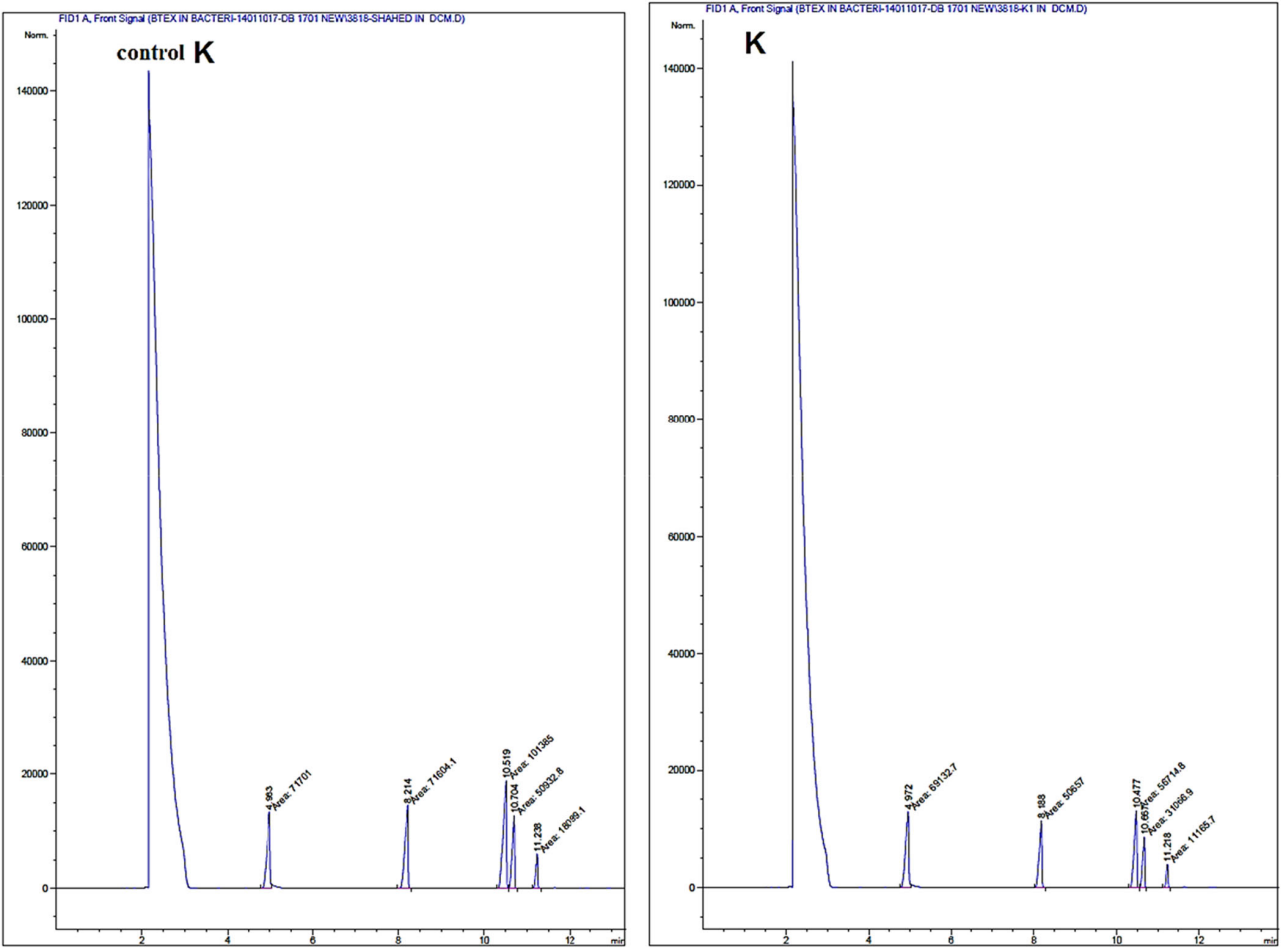
Chromatogram result from the biodegradation of BTEX by K bacteria along with the control sample through GC chromatography analysis based on the GC‐FID test method. The order of the peaks in the mentioned test, according to the column used, includes benzene, toluene, ethylbenzene, and total xylenes.

The gas chromatography method was employed to quantify the biodegradation of BTEX compounds by the selected bacteria. The results from the GC analysis is presented in Figure 4, with the corresponding chromatograms in Figures 5 and 6. The data indicate that G2 bacteria exhibit the highest biodegradation capacity, successfully decomposing and removing 84.57% of Toluene, 83.29% of Benzene, 83.03% of Xylene, and 82.55% of Ethylbenzene. M2 bacteria biodegraded and removed 84.53% of Benzene, 82.30% of Toluene, 74.18% of Xylene, and 74.09% of Ethylbenzene. In contrast, K bacteria demonstrated the lowest decomposition rates among those isolated, biodegrading 43.85% of Ethylbenzene, 35.13% of Xylene, 28.20% of Toluene, and only 4.87% of Benzene.

### 3.5 Identification of Selected Bacteria

The phylogenetic analysis of the best‐isolated strains showed that the bacterium named under the title G2 has 98.36% similarity to the *Arthrobacter pascens strain B46W* 16S ribosomal RNA gene, and the bacterium named under the title M2 has 99% similarity to the *Bacillus sp strain jrb‐12w*16S ribosomal RNA.

## 4 Discussion

BTEX hydrocarbons are a common contaminant in groundwater and other water sources, resulting from the disposal of contaminated industrial effluents and oil spills. Many studies have proven the presence of BTEX in drinking water (Fayemiwo et al. 2017). Zhang et al. (2012). demonstrated the presence of BTEX compounds in the air and their subsequent transfer from air to water. Biological degradation of BTEX compounds has been observed in contaminated sites under various environmental conditions. Among these, bacteria that have been exposed to BTEX for extended periods and have adapted to it play a crucial role (Fayemiwo et al. 2017). Wolicka et al. (2009). reported that the biodegradation of BTEX occurs more rapidly under aerobic conditions. In their study, Kim et al. (2008). successfully isolated 80 bacterial strains from gasoline‐contaminated soil samples. In this study, we isolated 12 aerobic bacterial strains from petrochemical waste samples and soil contaminated with oily sludge that had been exposed to petroleum and petroleum‐derived compounds for an extended period. Isolation was performed using chemotaxis, a rapid and efficient method. We then evaluated the potential of these bacterial isolates to remove BTEX. As indicated in Table 1, each bacterium exhibited movement toward a carbon source. In this study, the aim is to isolate bacteria that can consume and decompose the BTEX mixture. For this purpose, the growth and tolerance of the isolated bacteria in the presence of benzene, toluene, ethylbenzene, and xylene as carbon sources were investigated. Based on the investigation results, we selected seven bacterial strains that exhibited the highest growth rates in the presence of all four carbon sources. As shown in Table 2, while these selected bacteria preferred one carbon source during chemotaxis, they also demonstrated good growth with the other sources. Consequently, it shows their potential to biodegrade mixed BTEX compounds. Bacterial growth in the presence of carbon sources is the primary requirement for biodegradation. For this purpose, the turbidity measurement method or colony counting method is used in different studies. In the studies conducted by Handayani et al. (2019), the growth of *P. putida* and *B. cereus* was investigated in the presence of BTEX, and the results indicated that P. putida reached its logarithmic phase between 24 and 84 h of incubation, while *B. cereus* entered its logarithmic phase between 24 and 96 h. In the present study, we demonstrated through turbidity measurements and colony counting experiments that G2 (*Arthrobacter pascens*), M2 (*Bacillus sp*), and K bacteria exhibited the best growth in the presence of BTEX. The logarithmic growth phase of G2 bacteria is between 3 and 5 days with a maximum cell density of 78 × 10^8^ CFU/mL, M2 bacteria is between 5 and 7 days with a maximum cell density of 80 × 10^8^ CFU/mL and K bacteria is between 5 and 7 days with a maximum cell density of 5.8 × 10^8^ CFU/mL. As a result, these three bacterial strains, which demonstrated the best tolerance and growth rates in the presence of 1% BTEX, were selected for further studies to assess the biodegradation rate of BTEX. Singh and Celine (Singh and Celin 2010) demonstrated in their study that the Bb5 strain of *Pseudomonas sp*. exhibits the highest growth in the presence of BTEX compounds. GC analysis revealed that this strain can decompose 100% of benzene and ethylbenzene, 80% of toluene, and 70% of xylene. Furthermore, studies indicate that BTEX degradation varies among different bacterial species. Sarkar et al. (2013). evaluated the performance of *P. Putida* and *B. Cereus* in BTEX degradation, and the degradation rate increased in the order X < B < E < T. Shim et al. (2005) reported the need for periods of more than 5 days for the complete degradation of BTEX by *P. putida*. They also observed that the biodegradation rate by this bacterium was in the order E < X < B < T. In the present study, the time required for complete decomposition was found to exceed 10 days. The results indicated that the isolated bacteria were capable of degrading BTEX, although with varying efficiencies. In our studies, the chromatographic results and OD_600_ readings of residual BTEX dissolved in dichloromethane corroborated each other. The data indicated that the highest reduction of BTEX by our selected bacterial strains occurred in the following order: *Arthrobacter pascens* > *Bacillus sp*. > K. Additionally, as previously mentioned in the evaluation of these bacteria regarding their growth and tolerance in the presence of BTEX, the order of tolerance was G2 > M2 > K. This finding confirms that bacteria exhibiting high tolerance and growth in the presence of a carbon source also possess a greater ability to biodegrade that carbon source. Our results indicated that the G2 bacteria, known as *Arthrobacter pascens*, exhibited the highest efficacy in BTEX decomposition, successfully breaking down 83.29% of benzene, 84.57% of toluene, 82.55% of ethylbenzene, and 83.03% of xylene. Therefore, the percentage of BTEX compound degradation by *A. pascens* is in the order T > B > X > E. M2 bacteria, identified as *Bacillus sp*, were able to decompose 84.53% of benzene, 82.30% of toluene, 74.09% of ethylbenzene, and 74.18% of xylenes. Therefore, the order of percentage removal of BTEX compounds by the isolated *Bacillus sp*. is B > T > X > E. Finally, the lowest rate of biodegradation by bacteria isolated by us belonged to K bacteria known as *Actinomycete*, which was able to decompose 4.87% of Benzene, 28.20% of Toluene, 43.85% of Ethylbenzene and 35.13% of Xylene. According to the results, the percentage of BTEX compounds degradation by this bacterium is in the order E > X > T > B. Interestingly, *A. pascens* (G2) exhibited chemotactic movement towards ethylbenzene; however, it demonstrated the highest percentage of biodegradation with toluene and also showed significant biodegradation capabilities with other carbon sources in the BTEX group. The bacterium *Bacillus sp*. (M2) exhibited chemotaxis towards xylene, but it achieved the highest rate of biodegradation for benzene. In the case of K bacteria, there was movement toward ethylbenzene during chemotaxis, and the highest percentage of biodegradation also occurred with ethylbenzene. These results indicate that factors beyond the bacteria's biodegradation capability influence their movement toward the carbon source.

## 5 Conclusion

In this study, we aimed to identify the most effective native bacteria for BTEX decomposition that can significantly reduce this pollutant. We successfully isolated three bacterial strains that showed a strong ability to tolerate and grow in the presence of 1% BTEX. Then, the rate of BTEX degradation by these bacteria was evaluated through OD_600_ readings of residual BTEX dissolved in dichloromethane and chromatography. The results revealed that in the current study, *A. pascens* proved to be the most efficient bacterium in degrading BTEX, decomposing all four carbon sources within 12 days at a rate exceeding 80%. In BTEX biodegradation, *Bacillus sp*. bacteria ranked second, successfully removing more than 80% of benzene and toluene and over 70% of ethylbenzene and xylene within 12 days. Our findings support the use of *A. pascens* and *Bacillus sp*. bacteria in the bioremediation of sites contaminated with BTEX hydrocarbons. These bacteria demonstrate a high biological degradation capacity, which facilitates the development of a preferential pollutant treatment system.

## Author Contributions

**Zahra Mahmoodi Panah:** investigation. **Maryam Jalili Tabaii:** project administration, investigation, supervision. **Masumeh Sadat Shahidi Rizi:** project administration, writing – original draft, writing – review and editing, investigation, supervision.

## Ethics Statement

The authors have nothing to report.

## Consent

All authors approved the publication of this manuscript.

## Conflicts of Interest

The authors declare no conflicts of interest.

## Data Availability

The data supporting the findings of this study are available within the manuscript presented on the tables and graphs and figures in the articles. All data and materials of this study are available within the article.
